# A rare case of giant myopericytoma of the lower limb: a case report and review of the literature

**DOI:** 10.1016/j.ijscr.2025.111513

**Published:** 2025-06-14

**Authors:** Jianmin Cai, Le Xiao, Tengda Huang, Farong Li, Hongxia Lu

**Affiliations:** a Guangdong Medical University, Zhanjiang 524002, China; b Guangdong Medical University Shenzhen Baoan Clinical Medical College, Shenzhen Baoan District People's Hospital, Shenzhen 518101, Guangdong Province, China

**Keywords:** Myopericytoma, Ultrasonography, Magnetic resonance imaging

## Abstract

**Introduction and importance:**

Myopericytoma (MPC) is a rare soft tissue tumor that typically occurs in the extremities or distal limbs. These tumors are usually slow-growing, painless, and measure <2 cm on average. While generally benign, they can lead to complications if misdiagnosed. Surgical excision is the definitive treatment.

**Presentation of case:**

A 15-year-old female from southern China presented with a painless mass in her right thigh, which had persisted for three years. Imaging revealed a mass measuring approximately 8.5 cm, initially suspected to be a synovial sarcoma. Magnetic resonance imaging (MRI) showed a large, irregular soft tissue mass adjacent to the lower femur with heterogeneous signal intensity. Surgical excision was performed, and histopathological analysis confirmed the diagnosis of MPC.

**Clinical discussion:**

MPC is rare and often misdiagnosed owing to its nonspecific clinical and radiological features. This case is notable for the size of the mass, which exceeds the typical size of MPCs, making it the largest reported case of lower extremity MPC in Asia. A definitive diagnosis is made through histopathological examination, and surgical excision remains the gold standard treatment. Given its rarity, early recognition and accurate diagnosis are essential to avoid mismanagement.

**Conclusion:**

This case highlights the rarity of MPC and underscores the necessity of comprehensive diagnostic evaluation. Surgical excision is essential for symptom resolution and complication prevention.

## Highlights

• This report presents a rare case of a giant myopericytoma (8.5 cm) in the lower extremity, the largest in Asia.
• MPC diagnosis is challenging due to atypical size, deep location, and cyst-solid components mimicking other tumors.
• Surgical resection is the primary treatment for MPC, with histopathology as the diagnostic gold standard.
• The rarity and imaging nonspecificity of MPC necessitate long-term clinical follow-up.

## 1 Introduction

MPC is an exceedingly rare perivascular tumor composed of myoid cells, exhibiting histological features resembling both myocytoma and hemangiopericytoma. MPC predominantly affects adults and is most commonly located in the distal limbs. It was first reported by Granter [1] in 1998. MPC can occur at any age and in individuals of any gender. MPCs in soft tissues typically measure <2 cm in diameter [2]. Clinically, they primarily manifest as dermal or subcutaneous soft tissue tumors located in the proximal limbs, neck, trunk, and mouth. These tumors are usually slow-growing and painless [3].

Imaging findings of MPC are often nonspecific. On ultrasound, MPCs typically appear as hypoechoic masses with either well- or ill-defined margins, heterogeneous internal echoes, and peripheral capsular echoes in some areas. Color Doppler flow imaging (CDFI) often reveals abundant intralesional blood flow signals. In contrast, MRI demonstrates masses exhibiting prolonged T1-weighted and T2-weighted signal intensity, with more pronounced capsular enhancement. The fat-suppression sequence suggests minimal fat content within the mass [[4], [5], [6], [7]]. We report a challenging case of MPC in which preoperative imaging studies, while detecting the mass, failed to provide a definitive diagnosis of MPC. The patient underwent a successful surgical excision. To our knowledge, this is the largest reported case of MPC in Asia to our knowledge. The postoperative recovery was favorable, highlighting the significance of this case report.

## 2 Case presentation

**Fig. 1:**
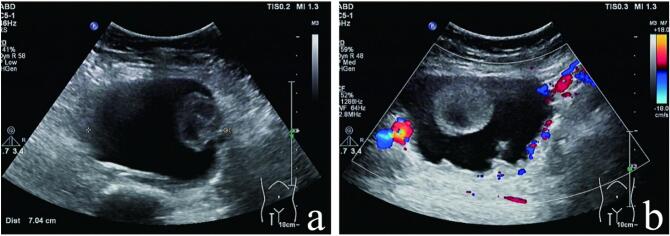
(A) Two-dimensional ultrasonography of a mixed echogenic mass in the muscular layer of the right thigh: A two-dimensional ultrasonography revealed a mixed echogenic mass measuring 8.5 × 7.3 × 5.3 cm in the muscularis propria of the middle and lower right thigh. The mass had clear boundaries but displayed an irregular morphology. It appeared mostly anechoic with an irregular wall. Additionally, a rounded and slightly strong echogenic mass measuring approximately 3.5 × 2.2 cm was observed attached to the capsule; (B) Color Doppler flow imaging of a right thigh mass: the mass did not exhibit any blood flow signal on color Doppler flow imaging, but peripheral blood flow signals were observed in dots and stripes.

A 15-year-old female patient from southern China presented with a primary complaint of a mass in the right thigh. The onset of symptoms occurred in 2019 and persisted for three years. During her visit on September 5, 2022, she reported that the lump had increased in size over the past month. The patient denied a history of trauma or surgery and was otherwise in good health. Physical examination revealed a soft, well-circumscribed, and mobile mass measuring approximately 8 × 7 cm in the mid-to-lower right thigh. The overlying skin was smooth and intact without signs of redness, swelling, or rupture. Upon admission, a series of relevant examinations were performed, including blood routine, liver and renal function tests, and electrocardiogram, all of which were unremarkable. Tumor markers such as AFP, CEA, CA-125, CA-153, and CA-199 were within normal limits. Ultrasonography (Fig. 1) demonstrated a substantial mass within the muscularis propria of the lower right thigh, indicating a possible choroidal tumor or synovial sarcoma.

**Fig. 2:**
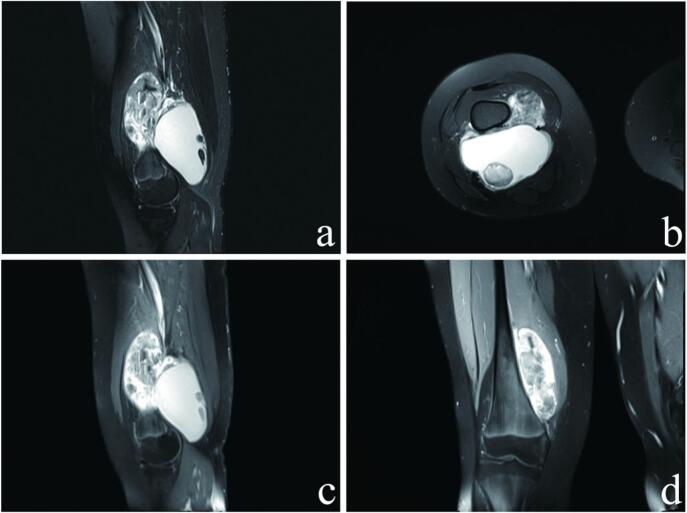
(A) Sagittal PDWI fat-suppressed sequence: The sagittal section under the PDWI fat-suppression sequence clearly depicts the tumor site. A large irregular mass is visible near the lower part of the femur, exhibiting mixed signals internally. (B) Axial PDWI fat-suppressed sequence: The cross-section under the PDWI fat-suppression sequence reveals a flaky mass on the left side of the femur, displaying both high and low signal intensities, with distinct boundaries. (C) Sagittal T1WI contrast-enhanced scan: The T1WI sagittal contrast-enhanced scan demonstrates significant heterogeneous enhancement of the cyst wall and solid components within the lesion, while no enhancement is observed within the cystic area. (D) Coronal T1WI contrast-enhanced scan: The T1WI coronal contrast-enhanced scan revealed a distinct demarcation between the mass and the cortical bone, indicating that the tumor is expanding within the soft tissue rather than infiltrating the femur.

Fig. 2 demonstrated an abnormal mass in the lower right thigh, suggestive of synovial sarcoma.

**Fig. 3:**
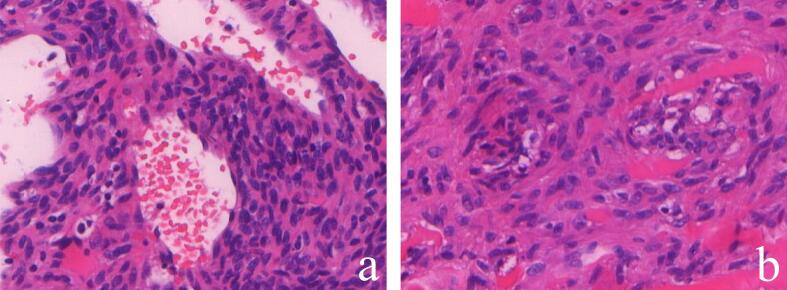
(A) Postoperative pathology section of perivascular spindle-shaped ovoid cells: microscopic view (HE, ×200): The tumor is observed in the perivascular region and is composed of spindle-shaped ovoid and myxoid cells. These cells have eosinophilic cytoplasm, homogeneous nuclear chromatin, inconspicuous nuclear anisotropy, and rare nuclear schizophrenia; (B) Postoperative pathology section of concentric tumor cell growth around vessels: microscopic examination (HE, ×200) reveals spindle-shaped or ovoid tumor cells exhibiting multilayered concentric circle-like growth patterns surrounding the blood vessels.

Following comprehensive patient consultation, surgical intervention was deemed necessary. The operation was performed on September 16 under general anesthesia, with successful excision of the affected soft tissue in the right lower limb. Intraoperative findings revealed that the tumor comprised a substantial amount of dark red turbid fluid and solid components exhibiting a gray-brown, fish-flesh-like morphology. The postoperative pathological examination (Fig. 3) definitively diagnosed the diagnosis of MPC. In addition, this work was reported in line with the SCARE criteria [8].

## 3 Discussion

MPC is a rare type of soft tissue tumor, typically not exceeding 2 cm in size (Supplementary Table 1). This article presents a remarkable case of a giant MPC located in the lower limb. Preoperative radiological examination demonstrated the tumor's localization within the deep muscular layer of the right thigh, immediately adjacent to the femur, and measured 8.5 × 7.3 × 5.3 cm, which is four times larger than the average tumor size previously reported in the literature. The patient underwent successful surgical treatment and remained asymptomatic without evidence of tumor recurrence during the 12-month postoperative follow-up period. Given the rarity and atypical manifestations of MPC, we aim to provide a concise overview of this disease. Our objective is to reduce the likelihood of misdiagnosis in future cases.

### 3.1 Clinical information of MPC

Clinical Manifestations: MPC predominantly manifests in the extremities, particularly in the lower limbs and distal regions. Clinically, it is characterized by slowly growing, painless nodules, with disease progression potentially spanning several years. However, some lesions may induce localized pain or discomfort. Most MPCs exhibit benign growth patterns, demonstrating a low risk of malignant transformation. Classic MPC is typically situated in the subcutaneous tissue, with deep soft tissue involvement being uncommon. And lesion size generally does not exceed 2 cm in diameter [2,9].

Diagnostic Methods: Patients commonly seek medical attention due to the presence of a palpable mass. Although imaging studies exhibit high sensitivity for detecting MPC, the lack of pathognomonic features complicates differentiation from other soft tissue tumors. Consequently, definitive diagnosis usually relies on postoperative pathological examination. Initial imaging may localize the lesion and preliminarily assess its basic characteristics, including size, boundary, and morphological features.

Histopathological Manifestations: Histopathological examination remains the gold standard for MPC diagnosis. Macroscopically, MPC appears as a well-circumscribed mass with a grayish-white or grayish-red surface. Microscopic evaluation reveals oval and short spindle-shaped tumor cells arranged concentrically around vascular structures, forming densely interwoven bundles of myoid cells. Immunohistochemical analysis demonstrates diffuse positivity for SMA and H-caldesmon, with focal Desmin expression. Notably, tumor cells typically lack expression of CK, CD34, CD31, and S100 protein [10,11].

Treatment and Prognosis: MPC typically does not resolve spontaneously, and surgery is often the preferred treatment modality. Postoperative outcomes are generally favorable, with rare occurrences of recurrence. Malignant MPC necessitates adjuvant radiotherapy and chemotherapy; however, the efficacy of these treatments remains uncertain and requires validation through further clinical case studies. Continuous follow-up is essential.

### 3.2 Diagnostic challenges of atypical MPC

The majority of classic MPCs are typically found subcutaneously and are rare in deep soft tissues. They usually do not exceed 2 cm in diameter. In this report, we present a case of a lesion located in the right lower extremity within the longissimus contractus muscle, which is a deep soft tissue. To the best of our knowledge, this is probably the largest case of lower extremity MPC found in Asia. Among the cases of lower extremity MPC that have been reviewed so far, tumors of this size are very rare.

MPC can be visualized through ultrasound examination, typically presenting as a regularly shaped hypoechoic solid mass [12]. However, in this case, the mass exhibited an irregular shape with mixed cystic and solid components. Postoperative examination confirmed that the cystic component was actually an old hemorrhage. Consequently, the irregular tumor morphology, combined with the presence of organized hemorrhage, complicated the diagnosis of MPC. Previous literature has reported a case of a giant MPC in the lower limb, where over 90 % of the tumor consisted of necrotic and hemorrhagic components [13]. The large size of the MPC may be associated with tumor liquefactive necrosis and accelerated growth due to late-stage hemorrhage. Due to these atypical manifestations, many physicians initially misdiagnosed the condition as other soft tissue tumors.

### 3.3 Differential diagnoses

Imaging examination is highly sensitive in diagnosing MPC, but it lacks specific characteristics and can be challenging to differentiate from other soft tissue tumors. Definitive diagnosis of MPC relies on pathology and immunohistochemistry. MPC needs to be distinguished from the following diseases:

Synovial sarcoma: This type of sarcoma is more common in young adults, particularly males. It frequently occurs in periarticular regions, particularly around the knee joint. Ultrasonographic imaging typically reveals lobulated hypoechoic masses with ill-defined margins, often accompanied by short linear or rod-shaped intralesional vascular signals on Doppler evaluation. Histopathological differentiation relies on characteristic immunohistochemical markers, with most cases demonstrating positive expression of CD99, BCL-2, and TLE1 proteins.

Synovial hemangioma: This rare benign soft tissue tumor occurs in children and adolescents and originates from the synovium. It usually affects the knee joint, causing pain and discomfort. Ultrasonography typically reveals hypoechoic masses with fluid dark areas. Color Doppler ultrasound may occasionally show low-velocity blood flow signals in solid masses. MRI examination is particularly valuable for diagnosing synovial hemangioma, as it demonstrates sinusoidal signals in T1WI, high signals in T2WI, and mixed signals of low, medium, and high due to different pathologic types. Enhanced scans of the lesions show moderate or obvious inhomogeneous enhancement. Thrombus and venous stones can be seen in some cases of synovial hemangioma [14,15].

## 4 Conclusions

MPC, a rare soft tissue tumor, is frequently misdiagnosed preoperatively due to its atypical clinical manifestations and imaging features, leading to delayed treatment. Clinicians and radiologists should maintain heightened vigilance for MPC, particularly when evaluating large (>2 cm), deep-seated, or cystic-solid masses in the extremities, as these may indicate atypical variants. To address diagnostic challenges, we recommend integrating advanced imaging (e.g., contrast-enhanced MRI) with histopathology and immunohistochemistry (prioritizing markers like SMA and h-caldesmon) to confirm pericytic differentiation. While the majority of myopericytomas demonstrate benign biological behavior, the potential for local recurrence and the rare occurrence of metastases in atypical and malignant variants necessitate careful postoperative monitoring, even following complete surgical resection. To ensure early detection of any disease recurrence, we recommend implementing a structured MRI surveillance protocol consisting of: an initial scan at 3 months postoperatively, followed by a second scan at 6 months, and subsequent annual examinations thereafter [16]. Future research should focus on elucidating the molecular drivers of MPC heterogeneity and validating novel biomarkers to refine diagnostic accuracy and explore targeted therapies. Collaborative multicenter studies are needed to establish evidence-based guidelines for managing giant or atypical MPCs, ultimately improving clinical outcomes for this underrecognized entity.

## Declaration of competing interest

None. All authors declare no conflicts of interest, financial or personal, that could influence the objectivity of this work.

## Appendix A Supplementary data

Supplementary material Image 1

## References

[1] Granter S.R., Badizadegan K., Fletcher C.D. (1998). Myofibromatosis in adults, glomangiopericytoma, and myopericytoma: a spectrum of tumors showing perivascular myoid differentiation. Am. J. Surg. Pathol..

[2] Alqassab A.T., Alsadah F.Z., Elsharkawy T., Alhamad M., Alsayed H. (2022). Ankle myopericytoma: a rare case report and cytogenetic study. Cureus J. Med. Sci..

[3] Iosue H., Rosenblum B. (2019). Myopericytoma of the foot: a case report. J. Foot Ankle Surg..

[4] Choi G.W., Yang J.H., Seo H.S., Kim W.T., Lee M.J., Yoon J.R. (2016). Myopericytoma around the knee: mimicking a neurogenic tumour. Knee Surg. Sports Traumatol. Arthrosc..

[5] Kagoyama K., Makino T., Mizutani T., Shimizu T. (2020). Intravascular myopericytoma on the right dorsal foot. Eur. J. Dermatol..

[6] Idarrha F., Aznague Y., Fathlkhir Y., Demnati B., Guedi Omar A., Amine Benhima M., Abkari I., Saidi H. (2021). Forefoot myopericytoma: a case report and review of the literature. Int. J. Adv. Res..

[7] Valero J., Salcini J.L., Gordillo L., Gallart J., González D., Deus J., Lahoz M. (2015). Intravascular myopericytoma in the heel. Medicine.

[8] Kerwan A., Al-Jabir A., Mathew G., Sohrabi C., Rashid R., Franchi T., Nicola M., Agha M., Agha R.A. (2025). Revised surgical CAse REport (SCARE) guideline: an update for the age of artificial intelligence. Premier J. Sci..

[9] Provenzano D., Lo B.S., Belfiore M., Buffone A., Cannizzaro M.A. (2017). Foot soft tissue myopericytoma: case-report and review. Int. J. Surg. Case Rep..

[10] Valero J., Salcini J.L., Gordillo L., Gallart J., Gonzalez D., Deus J., Lahoz M. (2015). Intravascular myopericytoma in the heel: case report and literature review. Medicine.

[11] Squillaci S., Cecchetti D., Tallarigo F., Pontieri F., Filardo A.V. (2005). Myopericytoma-type perivascular myoma located in the soft tissue of the foot: a case report and review of the literature. Pathologica.

[12] Van Camp L., Goubau J., Van den Berghe I., Mermuys K. (2019). Myopericytoma of the base of the finger: radiological and pathological description of a rare benign entity. J. Hand Surg.-Am..

[13] Peters K., Caracciolo J.T., Henderson-Jackson E., Binitie O. (2018). Myopericytoma/myopericytomatosis of the lower extremity in two young patients: a recently designated rare soft tissue neoplasm. Radiol. Case Rep..

[14] Barakat M.J., Hirehal K., Hopkins J.R., Gosal H.S. (2007). Synovial hemangioma of the knee. J. Knee Surg..

[15] Slouma M., Hannech E., Msolli A., Dhahri R., Kouki S., Metoui L., Gharsallah I., Louzir B. (2022). Synovial hemangioma: A rare cause of chronic knee painSynovial hemangioma: a rare cause of chronic anterior knee pain. Clin Case Rep.

[16] Hodzic R., Hodzic M., Piric N., Karasalihovic Z. (2019). Clinicopathologic features in a case of intermuscular myopericitoma of thigh. Acta Myol..

