# Supplementary material for: A rare case of giant myopericytoma of the lower limb: a case report and review of the literature
Source: Int J Surg Case Rep. 2025 Jun 14;133:111513. doi: 10.1016/j.ijscr.2025.111513 (PMC12240088; doi:10.1016/j.ijscr.2025.111513)
Supplement: Supplementary file 1 — Supplementary material [file mmc1.docx]

**Supplementary Table 1.** Clinical features of reported lower limb MPC cases.

| Author | Age/Sex | Location | Size(cm) | Treatment | Outcome |
| --- | --- | --- | --- | --- | --- |
| Choi et al.[1](2016) | 61/M | In the posterolateral area of the right knee | 1.2×1.1×0.7 | Complete surgical resection | N/A |
| Alqassab et al.[2] (2022) | 59/M | In the anterior aspect of the distal left ankle | 1.5×1.3×1.0 | Complete surgical resection | 2-week follow-up,no recurrence |
| Iosue and Rosenblum.[3] (2019) | 61/F | The plantar hallux and the second toe. | Diameter approximately 0.7-cm sized | Complete surgical resection | 2-week follow up, symptom-free with no recurrences |
| Provenzano et al.[4] (2017) | 65/F | In the web second web space between the 2nd and 3rd toe of the right foot | 0.6×0.5 | Complete surgical resection | N/A |
| Paek et al.[5] (2011) | 45/F | In the subcutaneous tissue of the posterior side of the right lower leg | 0.9×0.7 | Complete surgical resection | N/A |
| Agustí et al.[6](2016) | 63 /F | On her left foot sole | Diameter approximately 1-cm sized | N/A | N/A |
| Harish et al.[7](2006) | 68/M | Anterior to the Achilles tendon in Kager’s fat pad | 3.8×1.6×3.1 | Complete surgical resection | 15-month follow up, alive with no recurrence |
| Bellinger et al.[8] (2017) | 36/M | In the distal aspect of the femur | 3.3×2.2×1.0 | Complete surgical resection | 15-month follow up, alive with no recurrence |
| Idarrha et al.[9](2021) | 54/F | In the 2nd and 3rd inter-toe spaces | 2.0×2.0 | Complete surgical resection | 12-month follow up, alive with no recurrence |
| Terada.[10](2016) | 47/M | In the thigh | 4.0×3.0×3.0 | Complete surgical resection | 5-year follow up, alive with no recurrence |
| Ko et al.[11](2011) | 67/M | Right lateral thigh | Diameter approximately 3.5-cm sized | Complete surgical resection | N/A |
| Valero et al.[12](2015) | 48/M | On the heel of the right foot | 1.5×0.4 | Complete surgical resection | 12-month follow up, symptom-free with no recurrence |
| Hodzic et al.[13](2019) | 23/F | In the deep intermuscular tissue of her right lateral thigh. | 3.8×2.1×2.7 | Complete surgical resection | N/A |
| Cockburn et al.[14](2022) | 56/M | Right distal tibia, midline on his anterior leg. | 1.9×1.5×0. 9 | Complete surgical resection | N/A |
| Takeda et al.[15](2021) | 30/F | In the deep soft tissue of the popliteal region | 1.0×1.5 | Complete surgical resection | 6-month follow up, symptom-free with no recurrence |
| Khezami et al.[16](2021) | 16/M | In the anterior cruciate ligament | N/A | Complete surgical resection | 6-month follow up, symptom-free with no recurrence |
| Kagoyama.[17] (2020) | 71/M | On the right dorsal foot | 1.2×0.4 | Complete surgical resection | 12-month follow up, symptom-free with no recurrence |
| Rodrigues et al.[18] (2020) | 73/M | On the left knee | 1.2×0.6 | Complete surgical resection | 5-month follow up, symptom-free with no recurrence |
| Peters et al.[19]（2018） | 17/M | In the medial  left leg | 4.5×3.5×2.5 | Complete surgical resection | 2-month follow up, symptom-free with no recurrence |
| Peters et al.[19]（2018） | 22/M | Anterior to the  proximal tibia | 12.5×10.0×5.0 | Complete surgical resection | 2-month follow up, symptom-free with no recurrence |

References:

1. Choi GW, Yang JH, Seo HS, Kim WT, Lee MJ, Yoon JR: Myopericytoma around the knee: mimicking a neurogenic tumour. Knee Surgery, Sports Traumatology, Arthroscopy 2016, 24(9):2748-2751.

2. Alqassab AT, Alsadah FZ, Elsharkawy T, Alhamad MN, Alsayed H: Ankle Myopericytoma: A Rare Case Report and Cytogenetic Study. CUREUS J MED SCIENCE 2022, 0(2168-8184):0-0.

3. Iosue H, Rosenblum B: Myopericytoma of the Foot: A Case Report. The Journal of Foot and Ankle Surgery 2019, 58(4):811-813.

4. Provenzano D, Lo Bianco S, Belfiore M, Buffone A, Cannizzaro MA: Foot soft tissue myopericytoma: Case-report and review. INT J SURG CASE REP 2017, 41:377-382.

5. Paek JO, Kang HS, Yeo KY, Yu HJ, Kim JS: A Case of Myopericytoma on the Lower Leg. ANN DERMATOL 2011, 23(2):201.

6. Agustí J, Peñas L, Bosch N: Intravascular Myopericytoma of the Plantar Region: Case Report and Discussion of the Probable Origin From a Cutaneous Vascular Malformation. The American journal of dermatopathology 2016, 38(7):546-548.

7. Harish S, O Donnell P, Briggs TWR, Saifuddin A, Flanagan AM: Myopericytoma in Kager’s fat pad. SKELETAL RADIOL 2006, 36(2):165-169.

8. Bellinger E, John I, Zaccarini D, Damron TA: Osseous Myopericytoma Simulating a Giant Cell Tumor of Bone. JBJS Case Connector 2017, 7(2):e25.

9. Idarrha F, Aznague Y, Fathlkhir Y, Demnati B, Guedi Omar A, Amine Benhima M, Abkari I, Saidi H: FOREFOOT MYOPERICYTOMA: A CASE REPORTAND REVIEW OF THE LITERATURE. International Journal of Advanced Research 2021, 9(4):859-862.

10. Terada T: Myopericytoma of soft tissue (thigh). Human Pathology: Case Reports 2016, 3:37-40.

11. Ko JY, Choi WJ, Kang HS, Yu HJ, Park MH: Intravascular myopericytoma: An interesting case of a long‐standing large, painful subcutaneous tumor. PATHOL INT 2011, 61(3):161-164.

12. Valero J, Salcini JL, Gordillo L, Gallart J, González D, Deus J, Lahoz M: Intravascular Myopericytoma in the Heel. MEDICINE 2015, 94(11):e642.

13. Hodzic R, Hodzic M, Piric N, Karasalihovic Z: Clinicopathologic features in a case of intermuscular myopericitoma of thigh. Acta myologica 2019, 38(2):41-44.

14. Cockburn CJK, Crene EJD, Cockburn WJ: Pre-tibial myopericytoma: a case report. J SURG CASE REP 2022, 2022(2).

15. Takeda K, Nakamura M, Soda Y, Adachi N: Myopericytoma in the knee joint. Medicine: Case Reports and Study Protocols 2021, 2(7):e131-e136.

16. Khezami K, Gharbi A, Bassalah E, Jaafar S, Ganzaoui I, Bennour MA: Intra-articular leiomyoma of the knee mimicking a ganglion cyst in a child: A case report and review of literature. INT J SURG CASE REP 2021, 86:106320.

17. Kagoyama K, Makino T, Mizutani T, Shimizu T: Intravascular myopericytoma on the right dorsal foot. EUR J DERMATOL 2020, 30(1167-1122):736-737.

18. Rodrigues S, Dahlstedt-Ferreira C, Coelho TM, Gaspar D, Fallah J, Lino R, Geada N: Knee myopericytoma-case report and literature review. MOJ Orthopedics & Rheumatology 2020, 12(2374-6939):127-128.

19. Peters KB, Caracciolo JT, Henderson-Jackson E, Binitie O: Myopericytoma/myopericytomatosis of the lower extremity in two young patients: a recently designated rare soft tissue neoplasm. Radiology Case Reports 2018, 13(1930-0433):275-280.
